# Artificial Intelligence in the Diagnosis and Prognostic Stratification of Hepatocellular Carcinoma: Current Evidence, Clinical Applications, and Future Perspectives

**DOI:** 10.3390/biomedicines14030505

**Published:** 2026-02-25

**Authors:** Emily L. Pfahl, Nooruddin S. Pracha, Mohamed H. Emlemdi, Phuoc-Hanh D. Le, Mina S. Makary

**Affiliations:** 1 Department of Radiology, The Ohio State University College of Medicine, Columbus, OH 43210, USA; 2 Division of Vascular and Interventional Radiology, Department of Radiology, The Ohio State University Wexner Medical Center, Columbus, OH 43210, USA

**Keywords:** hepatocellular carcinoma, artificial intelligence, machine learning, deep learning, radiomics

## Abstract

The integration of artificial intelligence (AI) into medicine, oncology, and radiology represents a marked shift in the diagnosis, prognostication, and management of hepatocellular carcinoma (HCC), a malignancy with high global incidence and poor prognosis. This review examines the application of AI, including machine learning (ML) and deep learning (DL), across the spectrum of HCC care. As AI advances, new convolutional neural networks (CNNs) and other models are enhancing diagnostic accuracy, reducing interpretation times, and improving the characterization of liver lesions across major imaging modalities including ultrasound, computed tomography (CT), and magnetic resonance imaging (MRI). Beyond diagnosis, the transformative role of AI in prognostication is also improving, where AI can now noninvasively predict critical factors such as microvascular invasion, genetic mutation status, tumor recurrence, and treatment response. Furthermore, AI has shown promise in facilitating patient-specific treatment planning by stratifying patients for interventions such as transarterial chemoembolization (TACE) and stereotactic body radiation therapy (SBRT). The review also addresses the emerging fields of pathomics and the use of AI in positron emission tomography (PET), while critically evaluating the cost-effectiveness of these technologies. Despite its promise, the widespread clinical adoption of AI faces challenges, including limited generalizability, maintaining patient privacy, ethical considerations, and the need for robust prospective validation. Ultimately, this review illustrates that the future of HCC management lies in a collaborative, hybrid-intelligence model, where AI-driven insights augment clinical expertise to optimize diagnostic pathways, personalize therapy, and improve patient outcomes.

## 1. Introduction

The history of artificial intelligence (AI) can be traced back to the early 1950s with the advent of machine learning (ML), although its introduction into the field of medicine did not happen until the 1970s [1,2]. Growth plateaued in the mid-1980s but picked up exponentially with the invention of deep learning (DL) in 2000, which introduced the concept of artificial neural networks (ANNs) to simulate complex decision-making [1,3]. More recent advances in ML and DL have expanded the use of AI in the medical field, including in the diagnosis, treatment, and prognostication of diseases.

**Figure 1:**
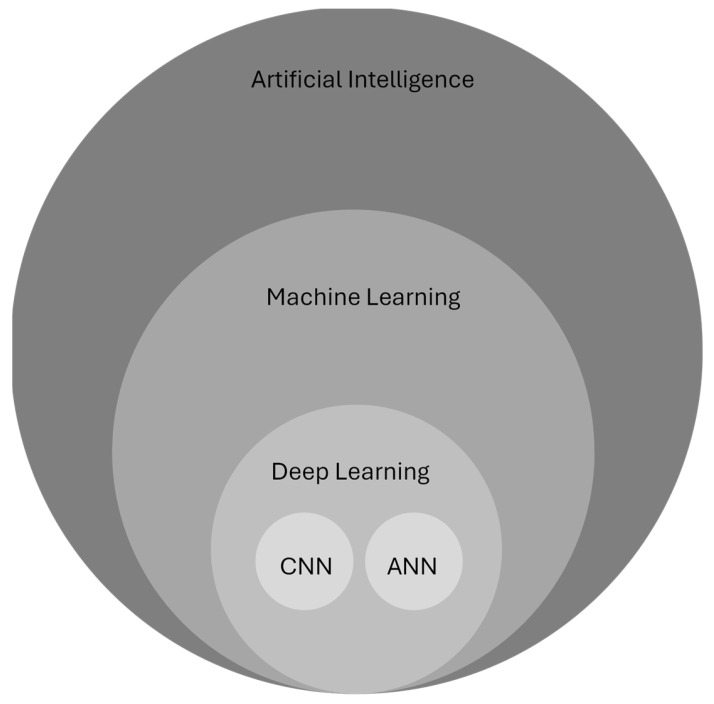
Relationship between artificial intelligence (AI) terminology. CNN = convolutional neural network; ANN = artificial neural network.

ML, DL, and ANNs, while related, are distinct topics. The narrowest focus of the three is ANNs, which are contained within the broader field of DL, which in turn is contained within the broadest field of ML [4]. All three concepts are contained within the larger framework of AI (Figure 1). At its most basic level, ML involves training computer programs to use data and models to make predictions about data patterns. DL uses neural networks to perform higher-order processing of data. ANNs, which comprise DL models, consist of multiple layers and are adept at pattern recognition and predictive modeling. Convolutional neural networks (CNNs) are similar to ANNs but are superior in image analysis and thus are highly valuable to the field of radiology [5].

The use of AI in the diagnosis of oncologic diseases has been widely studied, including its utilization in the diagnosis of lung, colorectal, skin, breast, and liver cancers [6,7,8]. ML algorithms have been used to diagnose cancer with a high degree of accuracy and have even been shown in some cases to outperform physicians [9]. Beyond diagnosis, predictive models have been used to identify patients at risk for developing cancers, as well as finding pre-cancerous lesions [10,11,12]. A new linear regression model, LORIS (logistic regression-based immunotherapy-response score), uses ML to predict cancer response to immune checkpoint blockade therapy, which can instruct clinical decision-making and improve treatment outcomes [13].

The intersection of AI and the field of oncology has been examined for a variety of malignancies, including hepatocellular carcinoma (HCC), a cancer with a yearly incidence of nearly one million cases worldwide [14]. In fact, HCC is the most common primary malignancy of the liver, accounting for about 85–90% of all liver malignancies with much of the rest being due to cholangiocarcinoma [15]. While viral hepatitis (specifically hepatitis B and hepatitis C) has historically been the predominant cause, the global burden of HCC is increasingly shifting toward non-viral etiologies [15]. In recent decades, targeted public health policies including universal hepatitis B vaccination and effective antiviral therapies have substantially reduced the incidence of virus-related HCC. Concurrently, the rising prevalence of obesity, metabolic syndrome, and type 2 diabetes has driven a dramatic increase in metabolic dysfunction-associated steatotic liver disease (MASLD), which has now emerged as the fastest-growing etiology of HCC worldwide [16]. Recent studies indicate that MASLD-related HCC incidence has increased by 2.3-fold over the past 15 years, now accounting for 10–20% of HCC cases in Western countries. This proportion is expected to increase [16]. The significant global burden of HCC demands increased attention and urgency for research in this area. Yet another recent study reported nearly 700,000 new cases of HCC and nearly 600,000 HCC-related deaths globally in 2022. The age-standardized rates of incidence and mortality are estimated to be approximately 6.8/100,000 and 5.9/100,000, respectively [17].

Furthermore, while HCC has classic imaging findings, diagnosis can be complicated by the presence of other pathologies, such as alcohol-related liver disease and MASLD. Though treatment options exist for HCC, prognosis remains relatively poor, with late-stage disease associated with a five-year survival rate of about 20% [18,19]. Through the application of predictive modeling using AI, HCC can be diagnosed earlier, and tumor characteristics can be used for disease prognostication. These models can also be used to predict patient outcomes and treatment response based on tumor and patient attributes, with the goal of improving survival [20,21,22]. Given how rapidly technology in medicine has been evolving, incorporating AI into the diagnosis, potential treatment, and prognostication of HCC has been a prudent next step in the evolution of AI-integrated medicine.

While existing literature has explored various other applications of AI in the realm of HCC, these reviews often focus on isolated aspects of implementation. A gap remains for a comprehensive synthesis that traces the full spectrum and role of AI, connecting its diagnostic capabilities directly to its impact on prognostication and personalized treatment planning. Additionally, the critical integration of emerging fields like pathomics and advanced PET analysis, alongside a pragmatic evaluation of the barriers to clinical translation, is often underemphasized. This review seeks to provide a comprehensive and clinically grounded overview of how artificial intelligence is shaping the care of patients with HCC. Rather than focusing on a single modality or application, we examine the role of AI across the entire spectrum of HCC management, from initial detection to prognostication and genetic correlation, to personalized treatment planning. We also explore emerging applications in pathomics and advanced PET imaging, areas where AI is beginning to unlock new insights beyond traditional imaging. Throughout, we pay particular attention to how these tools integrate with established clinical frameworks such as the Liver Imaging Reporting and Data System (LI-RADS), as building trust with clinicians requires demonstrating not just raw performance, but real-world utility alongside existing standards of care. Beyond technical performance, we discuss the practical realities of implementation, including cost-effectiveness, the limitations and biases inherent in current models, and the regulatory and ethical challenges that lie ahead. Finally, we look toward the future, considering what will be required to move these technologies from promising research into routine clinical practice; namely, prospective validation, greater model transparency, and the development of clear guidelines to ensure adoption that is safe and equitable.

Ultimately, this review illustrates that the future of HCC management lies in a collaborative, hybrid-intelligence model, where AI-driven insights augment clinical expertise to optimize diagnostic pathways, personalize therapy, and improve patient outcomes.

## 2. Methods of Literature Review Search

A literature search was conducted in the PubMed database to identify relevant articles published from 1 January 2019 until 31 October 2025. The following search terms and their combinations were used: “hepatocellular carcinoma”, “HCC”, “liver cancer”, “diagnosis”, “treatment”, “prognosis”, “prediction”, “artificial intelligence”, “AI”, “ultrasound”, “CT”, “MRI”, “PET”, “pathomics”, “radiomics”, “cost”, and “limitation”.

We included works published in the English language that addressed hepatocellular carcinoma and discussed artificial intelligence in relation to diagnosis, staging, treatment response assessment or prognosis prediction. Non-peer-reviewed works, abstracts, and articles published in a non-English language were excluded to maintain the integrity and comprehensibility of sources. The final determination of sources used was based on relevance, recency, and novelty of information.

**Figure 2:**
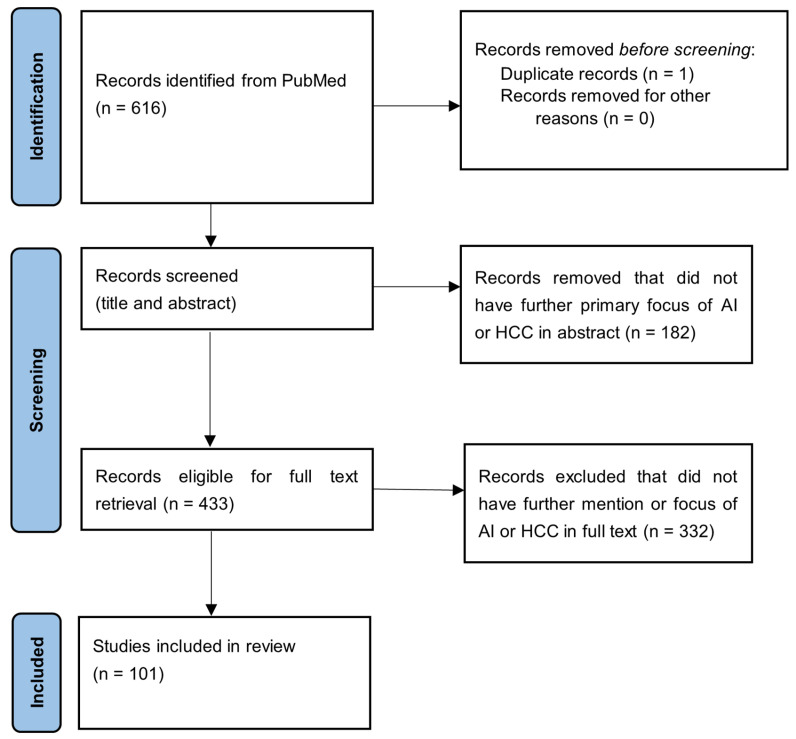
PRISMA flow diagram of study selection process.

The initial PubMed search yielded 616 records. Titles and abstracts were screened for relevance to hepatocellular carcinoma and artificial intelligence, and clearly irrelevant records were excluded. The full texts of the remaining articles were then assessed for eligibility, resulting in a final set of 101 publications included in this review. The study selection is depicted in the PRISMA flow diagram (Figure 2).

## 3. Advances in the Use of AI in Liver Cancer Diagnosis

The integration of AI, particularly DL models, into the field of oncology is rapidly advancing the diagnostic landscape of HCC, offering enhancements in accuracy, efficiency, and workflow across all major imaging modalities. Many AI models demonstrate exceptional diagnostic performance, reaching or even surpassing performance standards. To understand how accurate these models are, it is important to understand the area under the receiver operating characteristic curve (AUC), which is a performance metric that measures a model’s ability to distinguish between positive or negative classes, in this case, the presence or absence of disease. An AUC of 1.0 represents perfect classification, while an AUC of 0.5 represents performance no better than random chance. Many of the AI models reviewed exhibit high AUCs, making them increasingly important to consider for clinical practice. These tools are being developed not only to distinguish benign from malignant lesions with high specificity but also to classify specific tumor types, reduce radiologist interpretation time, and improve diagnosis of radiologically challenging cases. The following sections detail these advances as applied to ultrasound, computed tomography (CT), and magnetic resonance imaging (MRI).

### 3.1. Ultrasound and CEUS-Based AI Models

CNNs have been shown to diagnose HCC with high accuracy across multiple imaging modalities, including ultrasound, CT, and MRI. A comprehensive review of 183 studies of multiple modalities reported most models surpassing an AUC of 0.900 [23]. Conventional surveillance of HCC is primarily performed using B-mode ultrasound, which has multiple benefits including low cost, noninvasiveness, and high accuracy [24]. However, the sensitivity of ultrasound is limited by various factors, including operator dependency, poor penetration of certain tissues, and suboptimal detailing of deeper structures. This may necessitate further assessment by CT or MRI if ultrasound findings are inconclusive. One method for improving the diagnostic ability of ultrasound in focal liver lesions is the use of microbubble-based contrast agents; however, this technique is not currently recommended for the surveillance of HCC [25]. With room for improvement in surveillance, AI offers the opportunity to bridge the gap. A study of 49 patients investigated the combination of an AI model with contrast-enhanced ultrasound (CEUS) and found that the AI model achieved higher specificity (100% vs. 93.33%) but lower sensitivity (74% vs. 93.18% vs. 90.91%) in benign and malignant classification of liver lesions compared to two clinicians [26]. Advancing beyond benign and malignant classification, an algorithm trained with 367 liver samples was able to further classify liver lesions into subtypes with a mean AUC of 0.916 for focal liver lesion characterization and 0.931 specifically for classifying HCC [25]. These results show promise in the use of AI for surveilling HCC, especially in the realm of cost-effective ultrasound.

### 3.2. Other Imaging-Based AI Models

As a relatively low-cost and quick imaging option, CT plays an important role in HCC diagnosis. While ultrasound is the initial imaging study of choice for suspected HCC, CT with intravenous (IV) contrast is typically used to further characterize the extent of the lesion and for cancer staging [27]. Because HCC has unique radiographic findings, there is ample opportunity for AI integration. In multiple studies, the implementation of AI in CT resulted in significantly decreased radiologist read time [28,29,30]. AI can also improve the accuracy of diagnosis on CT by radiologists. Using a new CNN model to assess liver tumor burden, Vivanti et al. found that their methodology resulted in a diagnostic accuracy of 86% and had the ability to diagnose new tumors [31]. Studies of non-liver cancers suggest that AI may be helpful in diagnosing visually occult disease. One study found that AI could detect pancreatic cancer on CT before it could be seen with the naked eye [32]. In a retrospective study that used a radiomics-based classification model with 43 integrated features, the AI model outperformed radiologists in the diagnosis of HCC and intrahepatic cholangiocarcinoma with an AUC of 0.855 (vs an AUC of 0.689 for radiologists) [33]. Thus, the advances seen with CT further emphasize the ability of AI to make an impact on HCC detection and diagnosis.

The role of MRI in the diagnosis of HCC is typically to further characterize lesions or provide more detail when other imaging modalities are inconclusive. Similarly to its use in CT imaging, AI significantly decreases the reading time required for MRI cases. In one study of 100 prostate MRI scans, radiologists read cases with and without a computer-based diagnosis system. When using the DL-trained system, radiologists’ mean reading time decreased by 21% [34]. AI training models have also been shown to improve liver tumor diagnosis on MRI. Hamm et al. (2019) used a CNN-based DL model to diagnose and classify liver lesions on MRI; this model had a sensitivity of 90% for HCC diagnosis, compared to 60–70% for radiologists [35]. However, radiologists outperformed the CNN model on diagnosis of benign lesions and non-HCC malignancies [35].

One multicenter study of over 50,000 liver MRI images from nearly 2000 patients with liver tumors used a DL model to classify liver lesions. This model was able to accurately classify lesions as benign, malignant, or metastatic, with AUCs of 0.91, 0.873, and 0.876, respectively [36]. Sensitivities were also high for each lesion type, ranging from 88 to 98% [36].

Another proposed function of the use of AI in HCC diagnosis is improving workflow by aiding in liver segmentation. This is an essential step when diagnosing liver lesions as the involved segment(s) guides treatment and informs prognosis [37]. Liver segmentation and localization of lesions can be time-consuming and challenging; integration of AI can improve efficiency of workflow while preserving accuracy of diagnosis. Using fully automated liver segmentation, Winkel et al. (2020) were able to decrease the mean processing time from 219 s to 10 s while maintaining an intraclass correlation coefficient of 0.996 (Table 1) [38].

As the technology, software, and implementation of AI-based techniques are refined, there is significant evidence of programs’ potential for early detection of HCC and improved patient outcomes.

### 3.3. Pathology-Based AI Models

In addition to imaging-based diagnosis, AI has important applications in pathology-based diagnosis of HCC. A DL model has successfully been used to diagnose HCC and distinguish it from cholangiocarcinoma and metastatic cancer, with AUCs of 0.989 and 0.998, respectively [39]. Like its use in liver segmentation, AI programs can be used to efficiently segment histopathological slides for HCC diagnosis [40]. There is evidence that AI models may be able to diagnose HCC with an accuracy comparable to pathology subspecialists; one study found that their model diagnosed 100% of lesions accurately, which was significantly higher than junior, intermediate, and senior pathologists [41]. Taken together, this data supports the use of AI models in histopathological diagnosis of HCC and the potential for streamlined diagnosis.

### 3.4. Blood-Based and Non-Invasive AI Models

Non-invasive techniques are quickly gaining traction in the realm of HCC diagnosis. As with other diagnostic techniques, AI models have proven useful for earlier detection of HCC. One study that combined dynamic network biomarkers, an advanced form of DL, with a CNN to create a mouse model with chemically-induced HCC. This program was applied to genome sequencing and assessment of gene expression, and accurately identified pre-disease states, including *p53* activation [42]. Mass spectrometry can also be used to predict HCC development based on proteomic biomarkers. One study’s model was able to predict liver cirrhosis to HCC transformation with an AUC of 0.890. This model was also able to predict HCC 11.4 months on average prior to imaging diagnosis [43]. A separate mass spectrometry-based model had an average accuracy of 0.934 for HCC diagnosis [44]. There is evidence that non-invasive, non-imaging techniques, when combined with AI models, can successfully diagnose HCC and may be able to predict HCC development.

### 3.5. Correlation, Comparison, and Integration with Clinical Scoring Guidelines

To ensure the accuracy of novel ML algorithms, it is paramount to compare them to established clinical staging systems. LI-RADS is one clinical framework that is used to diagnose and stage liver lesions, including HCC. Studies with the intention of correlating intelligence models with LI-RADS have been conducted in three major imaging modalities: CEUS, CT, and MRI.

One study of 159 patients with LI-RADS category M nodules trained a radiomics and combined clinical model on CEUS images to differentiate HCC and non-HCC malignancies. They found that the radiomics and combined models performed better than the clinical model alone (AUC: 0.903, 0.912, and 0.698, respectively) [45]. A similar study showed higher accuracy using a random forest and generalized linear model to differentiate HCC and liver metastasis than LI-RADS alone [46]. This study also determined the most effective features for differentiating lesions on CEUS as mild and marked washout, unclear border, and rim enhancement [46]. Using a different ML program, Liang et al. (2025) were able to validate their predictive model by integrating CEUS features with LI-RADS, citing a concordance index of 0.804 at one year [47].

In a retrospective study of 26 patients with HCC and 23 patients without HCC, Okimoto et al. (2023) demonstrated improved LI-RADS interrater reliability with the assistance of a DL algorithm in CT [48]. A different retrospective study of 318 high-risk patients found that their ML program improved radiologists’ performance on diagnosing LR-5 lesions [49].

In their study using a radiomics model integrated with LI-RADS features, Liu et al. (2023) concluded that this hybrid model significantly outperformed eight other ML programs in MRI, achieving AUCs of 1.00 and 0.86 in the training and validation cohorts [50]. Another study using an integrated LI-RADS and DL model showed comparable accuracy to radiologists [51].

Aside from LI-RADS, other clinical systems exist for HCC diagnosis and prognostication. These include the Barcelona Clinic Liver Cancer, the Cancer of the Italian Liver Program, Hong Kong Liver Cancer, the Okuda system, and albumin-bilirubin grade. AI models should also be correlated with these systems to ensure diagnostic and prognostic accuracy. This can be accomplished by comparing radiologists’ applications of clinical systems with AI models’ performances. Concurrence between the two would provide evidence of the validity of these models.

### 3.6. Practical Clinical Implementation

The implementation of hybrid intelligence models into clinical practice must be relevant and feasible, and many examples demonstrate its utility. The combination of multimodal methods with clinician oversight can result in superior performance in detecting HCC. For example, a model with multimodal features including clinical, radiological, and immunological features resulted in enhanced detection compared to single-modal models [52]. This multimodal model demonstrated high AUC with both internal and external validation cohorts as well as enhanced performance specifically in HCC subpopulations, including alpha-fetoprotein-negative or small tumors. Such frameworks could be deployed in real clinical workflows to improve review of high-risk or uncertain cases.

While the development and performance of AI have been exploding, its adoption requires further study to understand its feasibility. A recent study assessed the efficiency of AI assistance incorporated into clinical workflow in the detection of HCC on CT. In this study, it was determined that AI could be seamlessly integrated into the reading of CT images and analysis of HCC foci [53]. The investigators reported that CT images can be automatically imported into their platform to output results. This may quickly assist in the planning of the next steps, such as for surgical planning. With well-demonstrated validation results and evidence of feasibility, it is essential to consider further efforts for implementing hybrid intelligence models into clinical practice.

## 4. AI Utility in the Prognostication and Treatment of Liver Cancer

**Figure 3:**
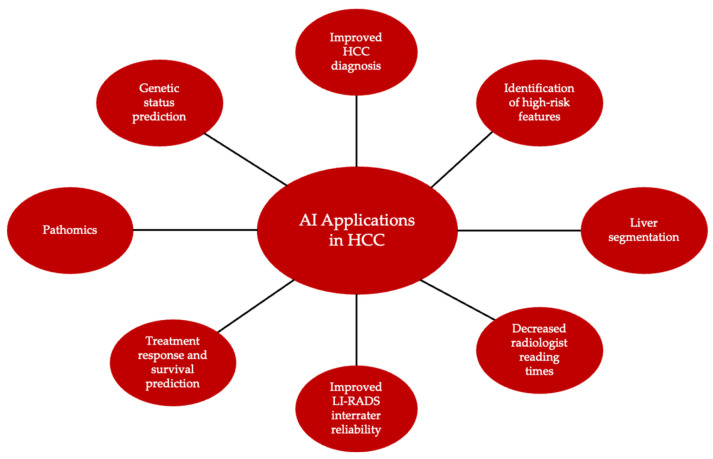
Summary of AI applications in HCC. HCC = hepatocellular carcinoma; LI-RADS = Liver Imaging Reporting and Data System.

Like other cancers, HCC prognosis is influenced by a variety of factors, including comorbid conditions, age, sex, genetics, and tumor stage. Though there have been advancements in treatment, the overall prognosis for HCC remains poor with a five-year survival rate of approximately 20%, making it the second-most lethal cancer [19]. Five-year survival rates are as high as 70% with curative treatment; however, delays in diagnosis limit treatment options, which decreases overall prognosis [54]. In addition to assisting with earlier diagnosis of HCC, AI can aid in the prognostication and recommendations for patient-specific treatment by analyzing tumor characteristics (Figure 3).

### 4.1. Ultrasound

While not the most sensitive imaging modality, ultrasound appears to still have value in the prognostication of HCC. Microvascular invasion is a prognostic factor that is negatively correlated with survival and positively correlated with early HCC recurrence [55]. In a study using an ANN trained to predict microvascular invasion, Wang et al. concluded that the model performed with high efficacy with an accuracy of 89% [56]. In another study, HCC recurrence was predicted using an AI model and ultrasound fusion imaging based on post-ablation features, with an accuracy of 85% [57].

Presence of the *TP53* gene is another poor prognostic factor that is associated with early tumor recurrence and metastatic disease. An ultrasound radiomics model created by Bu et al. was used to predict *TP53* mutation status in HCC based on tumor characteristics seen on ultrasound. One of the models was able to predict mutation status with an accuracy of 0.846 and an AUC of 0.823 [58]. When considering the low cost of ultrasound, augmentation with AI models makes it an attractive option for prognostication, especially in low-resource or underserved settings, where patients could most benefit from it.

### 4.2. Computed Tomography (CT)

While ultrasound is the gold-standard imaging modality for diagnosis of HCC, abdominal CT is superior in regard to monitoring post-treatment tumor response and disease prognostication due to its improved tissue characterization. In their study, Yao et al. focused on the prognostic factor of early tumor recurrence in patients who underwent partial hepatic resection. Using 180 patients as a training cohort, the authors created a model that predicted HCC recurrence on CT with high accuracy [59]. HCC stage is another prognostic factor that can be analyzed using AI in abdominal CT. An ML model applied to intermediate-stage HCC lesions post-TACE treatment was able to predict progression of disease to advanced-stage with an AUC of 0.97. The model was also able to successfully risk-stratify patients and determine which patients would benefit from systemic therapies [60].

Metastatic disease is a known poor prognostic factor for all cancer types, as it suggests aggressive disease with limited treatment options. Predicting which patients with HCC may develop metastatic disease based on tumor characteristics can therefore help guide treatment. He et al. investigated the use of a radiomics-based ML model in determining which HCC tumor characteristics on CT predict future metastatic disease. Their model, which combined features of HCC lesions with clinical risk factors, had significantly higher predictive potential when compared to the feature-based model and risk factor-based model alone. Using the model, key features of the tumors were shown to accurately predict the occurrence of metastatic disease overall [61].

As in ultrasound, AI models in CT can be used to apply genetic information to HCC prognostication. One study examined the association between *LOX* gene expression and HCC prognosis, as well as developed a model to predict *LOX* gene status based on tumor features on CT. A high expression of *LOX* in tumor tissue was associated with poorer outcomes, measured as a significantly reduced survival time. The radiomics model the study employed had good predictive value for tumor *LOX* expression with an AUC of 0.775 [62]. Collectively, these studies demonstrate that the integration of AI with CT imaging is transforming it from a purely anatomical tool into a powerful platform for prognostic prediction and personalized treatment planning in patients with HCC.

### 4.3. Magnetic Resonance Imaging (MRI)

Like CT, MRI is an imaging modality that can be used to characterize HCC response to treatment and analyze tumor features for prediction of prognosis. MRI offers superior soft-tissue contrast and multiparametric capabilities, combining conventional imaging with contrast-enhanced and functional sequences, allowing for a more comprehensive evaluation of the tumor microenvironment [63]. One recent study by Zhou et al. applied AI to contrast-enhanced MRI in HCC by using AI to calculate the tumor enhancement volume ratio (TEVR) from MRI [64]. They showed that the TEVR measured in the portal venous phase was strongly correlated with residual viable tumor on pathology (r = 0.89). Using a TEVR threshold of ≤19.5%, the model predicted a major pathological response with an AUC of 0.879. Tumors with an even stricter definition of ≤10% viable tumor cells corresponded to an even stronger reduction in recurrence risk (hazard ratio ~0.19). Yao et al. expanded on the value of longitudinal imaging, utilizing arterial phase MRI from multiple time points, including before TACE and following the first and second TACE sessions. Their model achieved an AUC of 0.92 and an accuracy of 0.86, outperforming radiomics-only models and the modified Response Evaluation Criteria in Solid Tumors criteria, an update to an older method for measuring treatment response based on tumor shrinkage. Through their approach, they were able to significantly stratify survival across risk groups [65].

MRI-based AI has demonstrated an emerging role in guiding individualized treatment strategies. Che et al. highlighted the unique opportunity MRI-based AI has to simultaneously integrate tumor and liver background features, emphasizing the capability to extract high-dimensional imaging features that correlate with molecular profiles and clinical outcomes, leading to more precise treatment planning. This study showed that these opportunities are not without limitations, namely the need for effective multimodal data integration. They underscore that future advancement will likely depend on an integrated approach, combining MRI with CT; genomic, transcriptomic, and pathological information; and longitudinal imaging to improve clinical relevance [66]. Across various treatment modalities including surgical resection, liver transplantation, TACE, and systemic therapies, AI-based MRI models have shown promise in predicting microvascular invasion, recurrence-free survival, and treatment response, thereby supporting personalized clinical decision-making. Furthermore, these models can noninvasively assess liver parenchymal status, such as fibrosis, and the risk of post-hepatectomy liver failure, which is critical for optimizing therapeutic strategies [66]. This collective progress underscores the transformative potential of AI-enhanced MRI to evolve from a diagnostic tool into a cornerstone of precision oncology for HCC.

### 4.4. Patient-Specific Treatment

Personalized treatment strategies are becoming increasingly important in managing liver cancer, as tailoring interventions to individual patient factors and tumor characteristics is critical for improving outcomes. A study by Zhenli et al. reviewed advances in predicting microvascular invasion (MVI), a key prognostic marker in HCC, and demonstrated that DL models outperform conventional methods in predicting preoperative MVI status. By accurately predicting MVI before surgery, clinicians can tailor treatment strategies regarding surgical resection margins, adjuvant therapies, or transplant eligibility to optimize outcomes for each patient. For instance, a comparative study found that a 3D-CNN DL model achieved an AUC of 0.906 for MVI prediction, slightly outperforming a radiomics-based XGBoost model (AUC: 0.887). Furthermore, accurate MVI prediction enables personalized perioperative management; a multicenter study showed that patients with MVI who underwent anatomical resection with a wide margin had significantly better median overall survival (OS) of 78.9 months compared to 51.5 months in those with narrow margins [67].

Expanding on how predictive approaches can allow for patient-tailored treatment, Dai et al. developed a CT-based deep learning radiomics scoring system to identify HCC patients likely to benefit from repeated TACE [68]. In their optimal model, which combined five radiomics signatures, DLscore and HBsAg demonstrated high predictive accuracy with AUCs ranging from 0.76 to 0.97. By accurately stratifying patients into high and low benefit groups, this model can enable clinicians to make individualized retreatment decisions, optimizing therapy for those most likely to respond to treatment while avoiding unnecessary interventions in others. Beyond TACE, AI models are also being developed to predict survival after SBRT. Chen et al. detail an interpretable ensemble model that combines radiomics and DL features from CT scans with clinical data to predict 2-year overall survival in HCC patients treated with SBRT. This ensemble model demonstrated a robust predictive capability, achieving an AUC of 0.86 (95% CI: 0.80–0.93), outperforming models based on clinical data alone (AUC: 0.71) or images alone (AUC: 0.59) [69].

In addition to CT-based models, MRI-based radiomics and DL approaches have shown promise in guiding individualized treatment. Xia et al. highlighted that HCC exhibits strong inter-tumor heterogeneity, which affects prognosis and response to therapy [70]. The study showed that image-derived metrics could stratify patients according to likely treatment response, helping guide decisions about surgery, TACE, or systemic therapy in a patient-specific manner.

Serial imaging over multiple treatment cycles can aid in identifying patients likely to achieve favorable response and longer survival. Xu et al. demonstrated this by developing a longitudinal CT-based radiomics model that predicts response and prognosis in patients with advanced HCC receiving immune checkpoint inhibitor-based therapy [33]. By capturing dynamic changes in tumor characteristics, this approach illustrates how AI can optimize clinical decision-making and tailor therapy to individual patients.

Selecting the right candidates for treatment is a key application of AI in personalized HCC therapy. Lin et al. developed an ML model based on 2.5D CT imaging to predict patient response to TACE [71]. The model provided an objective prediction of treatment efficacy prior to intervention. By stratifying responders from non-responders before TACE, clinicians are equipped with valuable information to guide patient-specific treatment decisions.

Together, these studies highlight how AI applied to CT and MRI can capture both static and dynamic tumor features, integrate data, and inform treatment selection in ways that were previously not possible. As these approaches continue to mature, the ability to personalize liver cancer management will increasingly depend on this multifaceted approach combining imaging with genomic, pathological and clinical information.

### 4.5. Positron Emission Tomography (PET)

While PET is not typically used as the first-line imaging modality to diagnose HCC, it does offer insight into disease prognosis, which can be augmented with the assistance of AI. MVI of HCC is a poor prognostic factor that is associated with early cancer recurrence and is a critical determinant of postoperative outcomes. One study using an ML-based logistic regression model was able to predict MVI with high accuracy [72]. In particular, radiomics analysis of [^18^F]FDG PET has demonstrated significant potential. A key study found that the maximum standardized uptake value (SUVmax) was the optimal parameter to predict MVI, achieving a sensitivity of 75%, specificity of 97%, and an AUC of 0.896 [67]. Other studies have used radiomic models to identify prognostic factors that successfully predict patient outcomes [73,74,75].

Beyond single metrics, comprehensive AI-derived risk scores are being developed for PET from multi-omics data to stratify patients. For instance, the artificial intelligence-derived risk score (AIDRS), constructed using a multi-step ML approach that integrated ten distinct algorithms, effectively predicted patient prognosis across multiple cohorts. A high AIDRS was significantly correlated with poor prognosis and a limited response to immunotherapy [76]. In addition to other imaging modalities, AI-automated liver segmentation can be used in PET for both HCC diagnosis and prognostication [75].

### 4.6. Pathomics

Pathomics, the application of AI to digital pathology, is emerging as a powerful tool in HCC management by enabling a more comprehensive analysis of tumor architecture and cellular morphology. Pathomics requires the extraction of quantitative features from histopathology images, capturing subtle patterns and morphologic changes beyond current means [77]. Still, there are obstacles such as variability in tissue staining, interpretability, and lack of standardized protocols limiting clinical adoption.

Spatial features in pathology, i.e., how cancer cells, stromal cells, and the broader tumor microenvironment are arranged relative to each other, are increasingly recognized as prognostic factors in HCC [78]. Yuan et al. demonstrated that, through the use of AI, spatial profiling of histology slides could stratify patients into distinct risk groups using patterns of tumor, stromal, and immune cell distribution to capture the intricate nuances and cellular heterogeneity [78]. This reflects the growing advances in and potential of using computational pathology in improving risk prediction and informing individualized therapy.

Another pathologic application of AI is ploidy assessment. Polyploidy is a condition in which cells have extra sets of chromosomes; if found in the cancer cells, this can be a poor prognostic factor for liver cancer. Matsuura et al. developed an AI system that could accurately distinguish polyploid tumors with poorer prognosis from other cells, something that currently requires specialized molecular testing [79]. By identifying more aggressive tumor phenotypes directly from standard pathology images, AI can enhance prognostic accuracy and help clinicians tailor surveillance and treatment decisions. Taken altogether, these developments illustrate how AI is bridging imaging, pathology, and clinical data to create a more integrated framework for precision oncology in HCC.

## 5. Cost Effectiveness and Privacy

### 5.1. Clinical Cost Effectiveness and Cost to Patients

The interplay of cost and benefit should be taken into account when adopting a novel technological advancement in medicine. While cost can refer to the money required to develop and operate a tool, the term also encompasses the impact on worker hours and productivity. AI models, when used to assist radiologists, can significantly decrease radiologists’ reading times for cancer diagnosis. One recent study found that radiologists’ hours were overall decreased by approximately half when using an AI model for HCC screening on ultrasound while also improving specificity and false positive rate [80].

Another example exploring cost-effectiveness of AI integration is apparent in a recent Italian healthcare study. The authors integrated AI with MRI scans for HCC surveillance. Their results showed only a marginal increase in cost of 5000 Euros per 1000 patients compared to the current standard of care in the region, while increasing quality adjusted life years (QALY) by 0.55 [66]. This yielded an incremental cost-effectiveness ratio of €9888 per QALY gained, which falls well below the Italian willingness-to-pay threshold of €33,000 per QALY gained, overall indicating strong cost-effectiveness [81]. This is promising in the context of a relatively low investment and is expected to improve as AI models mature and cost effectiveness increases [81]. This demonstrates that the implementation of AI models, starting with surveillance, is not only cost-effective, but clinically significant in the benefit they can provide to patients. Beyond this study, many others have shown a general increase in cost effectiveness of AI in medicine; however, further research is needed on more involved use of these models, especially in the context of HCC and for use in diagnosis, prognostication, and treatment of cancers [82].

### 5.2. Infrastructure Cost and Time Effectiveness

While the data on infrastructure costs of AI models and implementations specific to HCC are limited, either due to minimal reporting of these details or few scalable examples, the potential or expected costs can be extrapolated from adjacent fields of medicine. A 2024 study in the *Journal of the American College of Radiology* developed a comprehensive return on investment (ROI) calculator for AI-powered radiology platforms in general [83]. They demonstrated that implementing an AI platform in a stroke management-accredited hospital resulted in a 451% ROI over five years, increasing to 791% when radiologist time savings were fully accounted for [83]. Time savings included more than 15 eight-hour working days of waiting time, 78 days in triage time, 10 days in reading time, and 41 days in reporting time [83]. The calculator also captured downstream revenue benefits from clinically indicated follow-up scans and procedures, providing a framework applicable to HCC imaging workflows. While this study offers quantitative ROI data, it is also noted that results are sensitive to health center settings and scan volume, emphasizing the need for institution-specific modeling.

Another key infrastructure decision for facilities to make is in-house development versus opting for vendor solutions. This information again is limited in reference to HCC and for clinical uses of AI overall. However, generalized information currently can provide insight as further data from implementations is collected. Specifically, a 2024 study examined three implementation pathways for AI in healthcare including training from scratch (TSP), fine-tuned pathway (FTP), and out-of-the-box (OBP) [84]. Cost comparisons across major cloud service providers (Amazon, Microsoft, Google, Oracle) showed that TSP is the most resource-intensive regarding infrastructure and workforce while offering maximum customization and transparency [84]. FTP balances customization, cost, and performance, while OBP enables rapid deployment with minimal customization [84]. The authors emphasize that transitions between pathways may be necessary as health system needs evolve, and managed services can complement traditional workforce expertise.

A 2024 study in deep learning systems for intracranial hemorrhage detection in a large teleradiology practice found that system inefficiencies may outweigh benefits in high-volume, low-prevalence settings [85]. Examinations falsely flagged as positive by AI took significantly longer to interpret (9 min 40 s vs. 8 min 25 s at baseline, *p* < 0.001), demonstrating that implementation context and false-positive rates critically impact workflow efficiency [85]. This counterexample underscores the importance of rigorous local validation before deployment.

Returning to the context of HCC, current models must be further studied to see if they truly provide time effectiveness as this could vary widely based on real-world implementation.

## 6. Common Pitfalls and Limitations

One of the most obvious limitations of AI is the lack of generalizability. ML programs must be trained using a dataset. Because the translation of AI into clinical medicine has been more recent, there are limited studies on its use in HCC diagnosis and prognosis. A majority of models are trained on Caucasian patients and patients from the United States or China, which inherently introduces bias [86]. Patients of a different race or ethnicity than that of the training population may have inaccurate results, which may not be appreciated until after clinical deployment of the model [86]. Additionally, many studies use a relatively small patient population for program training, limiting the generalizability of results [87]. Along this line is the potential for the overfitting of data. If a model is trained to fit too tightly to the data on which it is trained, it may not be able to be accurately applied to other datasets. This is particularly a limitation when datasets are small.

Cancer diagnosis and prognostication is complex, requiring synthesis of patient history, imaging findings, clinical presentation, lab values, and genetic testing. Such complexity is outside the current capabilities of AI programs, which limits program applicability and accuracy in the setting of complicated tumors or rare presentations [88]. For example, HCC can present in noncirrhotic livers, have a significant fatty component, or have vessel invasion, which models may be unable to decipher [89].

Another limitation of AI in HCC imaging is misdiagnosis, especially when dealing with mixed or atypical tumor phenotypes. Differentiating HCC from other hepatic malignancies such as intrahepatic cholangiocarcinoma remains challenging, even for advanced models. A recent multicenter study developed an MRI-based radiomics model to distinguish dual-phenotype HCC from both conventional HCC and cholangiocarcinoma, which achieved high diagnostic performance [90]. However, confusion matrices demonstrated some misclassifications among tumor subtypes, reflecting the need for careful and continued validation before clinical integration.

Another important consideration is the cost to develop and implement an AI program in a healthcare system. While costs would be decreased in the long run [91], initial costs could pose a barrier to AI program development, particularly in community settings. An additional cost would likely be incurred by patients for the use of AI in their cancer diagnosis and prognosis, as is currently the case in optional AI-assisted mammogram readings, which appear to be more sensitive in diagnosing breast cancers [92,93]. This, however, leads to an ethical dilemma in which patients able to afford out-of-pocket fees for AI-enhanced readings are more likely to have their cancers diagnosed and therefore treated.

An anticipated barrier as AI becomes more integrated into healthcare systems is statewide regulations. There is currently no nationally established framework, although multiple societies at the local level are working to establish guidelines. According to the National Conference of State Legislatures, in 2024 forty-five states introduced AI-related bills to monitor, amend, limit, or ban the use of AI in public sectors, including healthcare [94]. Currently, Texas is the only state to pass a bill on the use of AI in clinical diagnosis, requiring providers to verify results before entering the information into patients’ charts. As other limitations of AI use in healthcare are addressed and programs become more widespread, it is expected that states will introduce bills with stricter regulations.

Patient and physician trust in the accuracy of AI models is another challenge in implementing the technology into healthcare settings. While not much research has been done in this regard, one vignette study of 860 patients in the Netherlands showed evidence that patients found physicians less trustworthy when using AI to make treatment decisions in high-risk cases, especially among women [95]. There are also various ethical concerns, including how patient data is used, inherent biases in training models, availability of AI based on patient socioeconomic status, and patient consent [96,97].

## 7. Future Directions

The integration of AI into the management of HCC has demonstrated substantial potential to enhance diagnostic accuracy, refine prognostic stratification, and guide personalized treatment. However, the full translation of this potential into routine clinical practice hinges on addressing several key areas in future research and development. The trajectory of AI in the context of HCC screening, diagnosis, and prognostication will be shaped by the creation of more sophisticated models, a deeper understanding of the human elements of implementation, and the establishment of robust frameworks for validation and regulation.

### 7.1. Improved Models

The next generation of AI models must evolve beyond single-task applications, such as tumor detection alone, and towards comprehensive, unified pipelines that manage the entire patient information journey from presenting symptoms to treatment recommendations [98]. This can possibly be achieved through the use of language models like ChatGPT-5.2, which can quickly compile complex patient medical histories [99]. Future AI models should focus on improved generalizability and robustness to overcome the current limitations of overfitting and performance degradation on data from different institutions or patient populations. This will require techniques designed to handle heterogeneous data to mitigate biases inherent in imbalanced datasets [98]. Furthermore, the complexity of models remains a significant barrier to clinical trust. Widespread adoption of Explainable AI (XAI) frameworks, such as SHAP (Shapley Additive Explanations) and LIME (Local Interpretable Model-agnostic Explanations), is therefore critical. Essentially, these frameworks would allow clinicians to comprehend the reasoning behind an AI’s output, transforming it from vague suggestions or pop-up messages into a transparent and actionable decision-support tool [100].

### 7.2. Patient Perception and Clinical Collaboration

The success of AI is not solely a technical challenge but also a human one. Patient perception and trust are pivotal for the adoption of AI programs. Current research suggests that trust in AI is highly dependent on its application; patients are most comfortable with AI in behind-the-scenes roles such as a diagnostic aid or digital scribe, and least comfortable with it replacing human interaction [100]. Ensuring transparency about AI use, guaranteeing human oversight, and enforcing strong data privacy protections are essential for building this trust. Ultimately, the future lies not in AI replacing clinicians, but in a collaborative, hybrid-intelligence model. This framework leverages the computational power of AI for data analysis and pattern recognition alongside the clinician’s expertise in complex decision-making, contextual understanding, and patient communication [98,100,101].

### 7.3. Need for Prospective Trials and Standardized Guidelines

Current AI models for HCC diagnosis lack clinical readiness due to absent external validation, prospective trials, and real-world deployment data. The majority of studies included in this review were single-center retrospective designs without independent cohort testing. No prospective randomized trials compare AI against standard clinical reading, and as of October 2025, no Food and Drug Administration- or Conformité Européenne-marked tools exist for HCC diagnosis.

For AI to move from research to widespread clinical practice, evidence from robust, multi-center prospective trials is indispensable. While numerous retrospective studies have shown promise, prospective trials comparing AI-assisted workflows directly against traditional care are needed to conclusively demonstrate improvements in hard clinical endpoints, such as overall survival, time to diagnosis, and cost-effectiveness [98,101]. The recent Italian cost-effectiveness study, which showed a favorable incremental cost-effectiveness ratio for AI-improved MRI surveillance of HCC, is a promising step in this direction. Parallel to this, the development of standardized guidelines for AI validation in hepatology is urgently required. Professional societies, such as the European Association for the Study of the Liver (EASL), are poised to establish these guidelines, which should define benchmarks for performance, require independent validation, and create dedicated sections in clinical practice guidelines for recommending validated AI models (Table 2) [98]. This will provide a clear regulatory pathway and ensure that tools are reliable, equitable, and safe for patient care.

## 8. Conclusions

Despite its current limitations regarding generalizability, data complexity, and initial implementation costs, AI has the potential to transform the healthcare landscape in which it is applied. There is promising evidence that AI models can be used to decrease radiologists’ read times, diagnose cancer at an earlier stage, and overall improve the efficiency of diagnosis and prognostication of HCC. As evidenced across ultrasound, CT, MRI, PET, pathology, and genomics, these tools are evolving from mere detection aids into sophisticated systems capable of predicting MVI, genetic mutations, and treatment response, thereby paving the way for highly personalized treatment plans. Realizing this full potential will require dedicated interdisciplinary collaboration to overcome existing hurdles. Clinicians, data scientists, engineers, and policymakers must work in concert to develop robust, explainable models, establish standardized validation guidelines, and create ethical frameworks that ensure equitable access. The future of HCC management lies in a synergy where AI-driven insights augment clinical expertise to ultimately improve patient survival and outcomes.

## Figures and Tables

**Table 1:** Summary of advancements in image-based AI models for liver cancer diagnosis and classification. CT = computed tomography; MRI = magnetic resonance imaging; AUC = area under the receiving operator characteristic curve; HCC = hepatocellular carcinoma.

Study	Modality	Objective	Measure	Result
Urhut et al. [26]	ultrasound	Benign vs. malignant classification	specificity	100%
			sensitivity	93.18%
Wongsuwan et al. [25]	ultrasound	Lesion subtype classification	AUC for focal liver lesions	0.916
			AUC for HCC	0.931
Vivanti et al. [31]	CT	Tumor burden assessment	Diagnostic accuracy	86%
Xu et al. [33]	CT	Diagnosis of HCC and intrahepatic cholangiocarcinoma	AUC	0.855
Hamm et al. [35]	MRI	Diagnosis and classification of liver lesions	sensitivity	90%
Zhen et al. [36]	MRI	Benign classification	AUC	0.91
		Malignant classification		0.873
		Metastatic classification		0.876

**Table 2:** Summary of future directions. HCC = hepatocellular carcinoma; AI = artificial intelligence.

Category	Next Steps for AI	Theorized Result of Implemented Steps on HCC Diagnosis or Prognostication
Improved Models	Ability to collect and synthesize patient healthcare information from medical charts	Better prediction of patient’s risk for HCC development, determination of probability of HCC diagnosis
	Increasing the generalizability of models	More robust analysis of patient images, increased accuracy, improved recognition of abnormal HCC presentations
	Explainable AI frameworks	Increased transparency of models, clinician and patient trust of AI reads
Patient Perception and Clinical Collaboration	Ensuring human oversight	Smaller chance of missed HCC diagnosis, clinician and patient trust of AI reads
	Data privacy protections	Increased comfort of patients and providers in using models, preservation of protected healthcare information
Need for Prospective Trials and Standardized Guidelines	Prospective trials	Quantification of the effect of AI integration on HCC diagnosis and prognostication
	Data on cost-effectiveness	Information on the feasibility of implementing AI models in different patient care settings
	Standardized guidelines	Ensured equitable, reliable, and safe patient care

## Data Availability

No new data were created or analyzed in this study.

## Abbreviations

The following abbreviations are used in this manuscript:

AI	Artificial intelligence
AIDRS	Artificial intelligence-derived risk score
ANN	Artificial neural network
AUC	Area under the receiver operating characteristic curve
CEUS	Contrast-enhanced ultrasound
CNN	Convolutional neural network
CT	Computed tomography
DL	Deep learning
FTP	Fine-tuned pathway
HCC	Hepatocellular carcinoma
HIPAA	Health Insurance Portability and Accountability Act
IV	Intravenous
LI-RADS	Liver Imaging Reporting and Data System
LIME	Local interpretable model-agnostic explanations
MASLD	Metabolic dysfunction-associated steatotic liver disease
ML	Machine learning
mRECIST	Modified response evaluation criteria in solid tumors
MVI	Microvascular invasion
OBP	Out-of-the-box
OS	Overall survival
PET	Positron emission tomography
QALY	Quality-adjusted life year
SBRT	Stereotactic body radiation therapy
SHAP	Shapley additive explanations
TACE	Transarterial chemoembolization
TEVR	Tumor enhancement volume ratio
TSP	Training from scratch
XAI	Explainable AI
